# Effects of short-term exposure to ambient airborne pollutants on COPD-related mortality among the elderly residents of Chengdu city in Southwest China

**DOI:** 10.1186/s12199-020-00925-x

**Published:** 2021-01-12

**Authors:** Jianyu Chen, Chunli Shi, Yang Li, Hongzhen Ni, Jie Zeng, Rong Lu, Li Zhang

**Affiliations:** Sichuan Provincial Center for Disease Control and Prevention, No.6, Zhongxue Road, Wuhou District, Chengdu, 610041 People’s Republic of China

**Keywords:** Air pollution, Sichuan Basin, COPD-related mortality, Elderly people

## Abstract

**Background:**

Chronic obstructive pulmonary disease (COPD) has become a severe global burden in terms of both health and the economy. Few studies, however, have thoroughly assessed the influence of air pollution on COPD-related mortality among elderly people in developing areas in the hinterland of southwestern China. This study is the first to examine the association between short-term exposure to ambient airborne pollutants and COPD-related mortality among elderly people in the central Sichuan Basin of southwestern China.

**Methods:**

Data on COPD-related mortality among elderly people aged 60 and older were obtained from the Population Death Information Registration and Management System (PDIRMS). Data on airborne pollutants comprised of particulate matter < 2.5 μm in aerodynamic diameter (PM_2.5_), sulfur dioxide (SO_2_), nitrogen dioxide (NO_2_), carbon monoxide (CO), and ozone (O_3_) were derived from 23 municipal environmental monitoring sites. Data on weather conditions, including daily mean temperature and relative humidity, were obtained from the Chengdu Meteorological Bureau. All data were collected from January 1, 2015, to December 31, 2018. A quasi-Poisson general additive model (GAM) was utilized to assess the effects of short-term exposure to airborne pollutants on COPD-related mortality among elderly people.

**Results:**

A total of 61,058 COPD-related deaths of people aged 60 and older were obtained. Controlling the influences of daily temperature and relative humidity, interquartile range (IQR) concentration increases of PM_2.5_ (43 μg/m^3^), SO_2_ (8 μg/m^3^), NO_2_ (18 μg/m^3^), CO (0.4 mg/m^3^), and O_3_ (78 μg/m^3^) were associated with 2.7% (95% CI 1.0–4.4%), 4.3% (95% CI 2.1–6.4%), 3.6% (95% CI 1.7–5.6%), 2.7% (95% CI 0.6–4.8%), and 7.4% (95% CI 3.6–11.3%) increases in COPD-related mortality in people aged 60 and older, respectively. The exposure-response curves between each pollutant and the log-relative risk of COPD-related mortality exhibited linear relationships. Statistically significant differences in the associations between pollutants and COPD-related mortality were not observed among sociodemographic factors including age, gender, and marital status. The effects of O_3_ remained steady after adjusting for PM_2.5_, SO_2_, NO_2_, and CO each time in the two-pollutant models.

**Conclusions:**

Increased concentrations of ambient airborne pollutants composed of PM_2.5_, SO_2_, NO_2_, O_3_, and CO were significantly and positively associated with COPD-related mortality in the central Sichuan Basin, which is located in the hinterland of southwestern China. The adverse effects of O_3_ were stable, a finding that should receive more attention.

**Supplementary Information:**

The online version contains supplementary material available at 10.1186/s12199-020-00925-x.

## Background

Chronic obstructive pulmonary disease (COPD) is characterized as a group of conditions that affect the structures within the lungs in various ways, including emphysema, asthma, chronic bronchitis, and others [1]. Recently, COPD has become a severe global burden in terms of both health and the economy and had become the fifth leading cause of mortality worldwide by the early twentyfirst century [2]. Ambient air pollution, as another severe health burden, now accounts for 1.2% of premature deaths and 0.5% of lost years of life worldwide [3]. Previous studies have reported air pollution and COPD incidences, hospital admissions, and mortality [4–7]. In China, most previous investigations concerning air pollution and COPD-related mortality were conducted in developed areas, including eastern coast regions, as well as Beijing and its surrounding areas [8, 9].

In the hinterland of southwestern China, where economic conditions are comparatively underdeveloped, few similar studies have been undertaken. Some previous investigations of this areas reported associations between air pollution and respiratory or cardiovascular morbidity and mortality [10, 11], but few presented associations between air pollution and COPD-related mortality. Accordingly, there remains a lack of information regarding the influence of air pollution on COPD-related mortality in the hinterland of southwestern China. In light of previous research, we conducted this study to (1) explore the effects of short-term exposure to ambient airborne pollutants (PM_2.5_, SO_2_, NO_2_, CO, and O_3_) on COPD mortality among elderly people living in the developing areas of southwestern China and (2) determine the sociodemographic factors (e.g., age, gender, and marital status) that modify the effects of pollutants on COPD-related mortality.

We conducted our study in Chengdu city, located in the center of the Sichuan Basin in the hinterland of southwestern China [12]. Chengdu experiences severe air pollution [13]. In addition, being the bottom of a basin, the diffusion of airborne pollutants in Chengdu differs from that of the plains in which previous studies were carried out in China. Chengdu is densely populated, with more than 16 million residents in 2017 [14]. The aforementioned advantages make Chengdu a suitable location for such a time-series study. To our knowledge, no similar reports concerning the associations between short-term exposure to ambient airborne pollutants and COPD-related mortality in this area have been presented. Thus, our study may add some limited evidence to this knowledge gap. An epidemiologically designed time series was conducted to assess the effects of short-term exposure to ambient airborne pollutants on COPD-related mortality among elderly people in Southwest China.

## Methods

### Data collection

Mortality data were obtained from the Population Death Information Registration and Management System (PDIRMS), which covers all the mortality information of residents in Chengdu city. After a resident was confirmed to have died by doctors in a hospital or at the resident’s home, his or her personal information regarding the death would be recorded in the system. The personal information consists of ID number, name, gender, age, date of birth, date of death, marital status, residential address, primary death diagnosis, secondary death diagnosis, underlying cause of death diagnosis, and other data. Private personal data that could be associated with a particular person, such as name and residential address, were omitted, while age, gender, marital status, primary death diagnosis, secondary death diagnosis, and underlying cause of death diagnosis were maintained. All records from January 1, 2015, to December 31, 2018, were obtained from PDIRMS. A death record was maintained for further analysis when it fulfilled the following two criteria: (1) the age of the resident was 60 or older and (2) the death diagnosis was COPD. The International Classification of Diseases, 10th Revision (ICD-10), was used to diagnose COPD (ICD-10 code J40-J44).

**Fig. 1:**
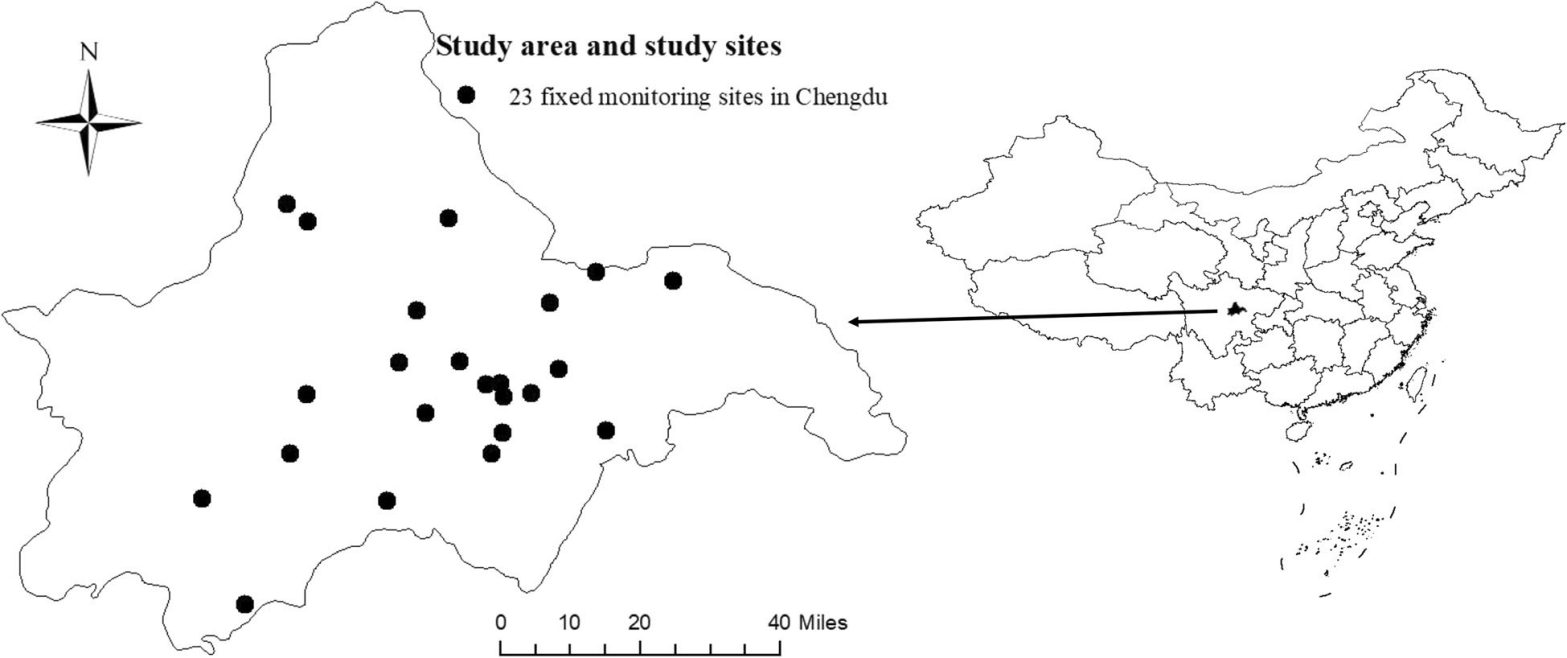
Location of study area in Chengdu city, southwestern China. The enlarged area on the left depicts the spatial distribution of 23 fixed monitoring sites throughout Chengdu city

Data on airborne pollutants comprised of particulate matter < 2.5 μm in aerodynamic diameter (PM_2.5_), sulfur dioxide (SO_2_), nitrogen dioxide (NO_2_), carbon monoxide (CO), and ozone (O_3_) were derived from 23 municipal environmental monitoring sites which operated continuously from January 1, 2015, to December 31, 2018, in Chengdu. These 23 sites were distributed almost evenly, covering both urban and rural areas in Chengdu (Fig. 1). Data for the real-time concentrations of each airborne pollutant were continuously monitored and recorded automatically 24 h a day at every fixed monitoring site. The daily mean concentrations of PM_2.5_ (μg/m^3^), SO_2_ (μg/m^3^), NO_2_ (μg/m^3^), and CO (mg/m^3^), as well as the daily 8-h mean concentrations of O_3_ (μg/m^3^) were calculated using data from all the sites. If the data from a given site were missing on a given day, data from the remaining sites were used to calculate the mean concentrations. The missing data for each day and each site were recorded for use in calculating the missing data rate of each pollutant. Weather conditions, including daily mean temperature and relative humidity, were obtained from the Chengdu Meteorological Bureau. These conditions were considered to be confounding factors and controlled in the models. Daily data on airborne pollutants and weather conditions were collected from January 1, 2015, to December 31, 2018.

### Statistical analysis

The correlations between airborne pollutants and weather conditions were explored using Spearman’s correlation test. Daily mortality, concentrations of airborne pollutants, and weather conditions were linked by date, correspondingly conforming to the design of the time series. Therefore, a quasi-Poisson general additive model (GAM) was utilized to assess the effects of airborne pollutants on COPD-related mortality. Daily mortality was set as the response variable, while the concentrations of airborne pollutants were the predictor variables in the model. A natural cubic spline function (NS) with degrees of freedom (DFs) was used to control the influence of time trends and potential nonlinear effects of confounding variables, including daily mean temperature and relative humidity. Based on previous studies [15], a total of seven DFs per year was selected for the time trends, while six DFs and three DFs were selected for daily mean temperature and relative humidity, respectively. Indicators of day-of-week (DOW) and holidays were included for weekly and holiday variations.

Delays and accumulations exist between air pollutant exposure and respiratory system health effects [16, 17]. Single-day lag models may underestimate the effects of airborne pollutants on mortality [18]. Multi-day lag models with different lag periods were employed to evaluate the cumulative effects from the various concentrations of each pollutant in our study, including the moving average concentrations of the case day and lags of up to 3 days (lag01, lag02, and lag03, respectively). The exposure-response curves were plotted in order to graphically depict the relationship between each pollutant and the log-relative risk of COPD-related mortality. The curves corresponded to the days of greatest cumulative effects for each pollutant.

In order to assess the modification of the effects by sociodemographic factors, different sociodemographic groups comprised of gender groups, marital status groups, and age groups were utilized. The gender groups and marital status groups were each stratified into two sub-groups, male and female, and married and alternative marital status (including divorced, widowed, and never married), respectively. The age groups were stratified into four sub-groups, 60–69, 70–79, 80–89, and ≥ 90 years.

*Z* tests were utilized for testing the statistically significant differences of effect estimates between effect modifiers of sociodemographic factors (e.g., the difference between “marriage” and “alternative marital status”). The equation is as follows:

$$ \mathrm{Z}=\left({\hat{Q}}_1-{\hat{Q}}_2\right)/\sqrt{S{\hat{E}}_1^2+S{\hat{E}}_2^2}, $$

where ${\hat{Q}}_1$ and ${\hat{Q}}_2$ are the estimates for the two categories, and $S{\hat{E}}_1$ and $S{\hat{E}}_2$ are their respective standard errors [19].

Two types of sensitivity analyses were conducted in order to test the robustness of the results. First, two-pollutant models were run for each pollutant by adding the remaining pollutant each time into the model. Second, alternative degrees of freedom (DFs) for time trends by year ranging from 5–9 were used in the models.

The potential adverse effects from airborne pollutants were calculated as relative risk (RR) with a 95% confidence interval (CI) for an interquartile range (IQR) increase of each pollutant. The equation was as follows:

$$ \mathrm{RR}=\exp \left(\beta \times \Delta X\right), $$

where *β* represents the estimate results from the GAM, and ΔX is the IQR for each pollutant.

The “MGCV” package in the R programming language (version 3.5.1) was used for fitting the time series model and plotting the exposure-response curves.

## Results

A total of 61,058 COPD-related deaths of people aged 60 and older were obtained from January 1, 2015, to December 31, 2018. Among these, 33,731 deaths were males and 27,327 were females. 31,948 of the deceased were married, while 29,110 had some sort of alternative marital status (including divorced, widowed, and never married). Deaths in the 60–69, 70–79, 80–89, and ≥ 90 age groups were 6527, 16,916, 27,781, and 9834, respectively. The mean value and IQR for each type of air pollutant, weather condition, and COPD-related death are listed in Table 1.

**Table 1:** Data for ambient air pollutants, weather conditions, and COPD-related deaths in Chengdu from 2015 to 2018

	Mean	SD	Min.	25%	50%	75%	Max.	IQR
PM_2.5_ (μg/m^3^)	58.7	38.9	8.0	31.0	47.0	74.0	254.0	43.0
SO_2_ (μg/m^3^)	14.5	6.3	4.0	10.0	14.0	18.0	41.0	8.0
NO_2_ (μg/m^3^)	40.5	13.1	13.0	31.0	38.0	49.0	92.0	18.0
CO (mg/m^3^)	0.9	0.3	0.4	0.7	0.9	1.1	2.2	0.4
O_3_-8h (μg/m^3^)	98.8	51.8	11.0	59.0	88.0	137.0	279.0	78.0
Temperature (°C)	16.7	7.3	− 1.9	9.9	17.3	23.0	29.8	13.1
RH (%)	80.8	8.9	41.0	75.0	81.0	88.0	99.0	13.0
COPD-related deaths	41.8	14.9	14.0	32.0	38.0	49.0	114.0	17.0
Age group (year)								
60–69	4.5	2.5	0	3.0	4.0	6.0	16.0	3.0
70–79	11.6	5.0	1.0	8.0	11.0	14.0	37.0	6.0
80–89	19.0	7.5	4.0	14.0	18.0	23.0	60.0	9.0
≥ 90	6.7	3.5	0	4.0	6.0	9.0	22.0	5.0
Gender								
Male	23.1	8.8	7.0	17.0	21.0	27.0	62.0	10.0
Female	18.7	7.5	4.0	13.0	17.0	23.0	61.0	10.0
Marital status								
Married	21.9	8.4	5.0	16.0	20.0	26.0	62.0	10.0
Alternative statuses ^a^	19.9	7.9	4.0	14.0	19.0	24.0	55.0	10.0

Abbreviations: *PM*_*2.5*_ particulate matter < 2.5 μm in aerodynamic diameter, *SO*_*2*_ sulfur dioxide, *NO*_*2*_ nitrogen dioxide, *CO* carbon monoxide, *O*_*3*_*-8h* daily 8-h mean concentration of ozone, *RH* relative humidity, *COPD* chronic obstructive pulmonary disease, *SD* standard deviation, *IQR* interquartile range

^a^Alternative marital statuses include widowed, divorced, and never married

The concentrations of the PM_2.5_, SO_2_, NO_2_, CO, and O_3_ ambient air pollutants exhibited typical seasonal tendencies. The concentrations of PM_2.5_, NO_2_, and CO were considerably higher during winter, and lower during summer each year, while the seasonal variations of the O_3_ concentrations were the opposite. The concentrations of SO_2_ exhibited weaker seasonal tendencies than the other pollutants, although the pattern of higher in winter and lower in summer was also apparent (Figure S1).

As shown in Table 2, correlations were observed among ambient air pollutants and weather conditions, except between SO_2_ and temperature, and between temperature and relative humidity.

**Table 2:** Spearman’s correlation coefficients of air pollutants and weather conditions

	PM_2.5_	SO_2_	NO_2_	CO	O_3_-8h	Temperature	RH
PM_2.5_	1						
SO_2_	0.540 ^a^	1					
NO_2_	0.799 ^a^	0.435 ^a^	1				
CO	0.820 ^a^	0.450 ^a^	0.712 ^a^	1			
O_3_-8h	− 0.215 ^a^	0.092 ^a^	− 0.227 ^a^	− 0.368 ^a^	1		
Temperature	− 0.511 ^a^	− 0.028	− 0.478 ^a^	− 0.551 ^a^	0.712 ^a^	1	
RH	− 0.109 ^a^	− 0.254 ^a^	− 0.118 ^a^	0.064 ^a^	− 0.510 ^a^	− 0.037	1

^a^*P* < 0.05

Abbreviations: *PM*_*2.5*_ particulate matter < 2.5 μm in aerodynamic diameter, *SO*_*2*_ sulfur dioxide, *NO*_*2*_ nitrogen dioxide, *CO* carbon monoxide, *O*_*3*_*-8h* daily 8-h mean concentration of ozone, *RH* relative humidity

**Fig. 2:**
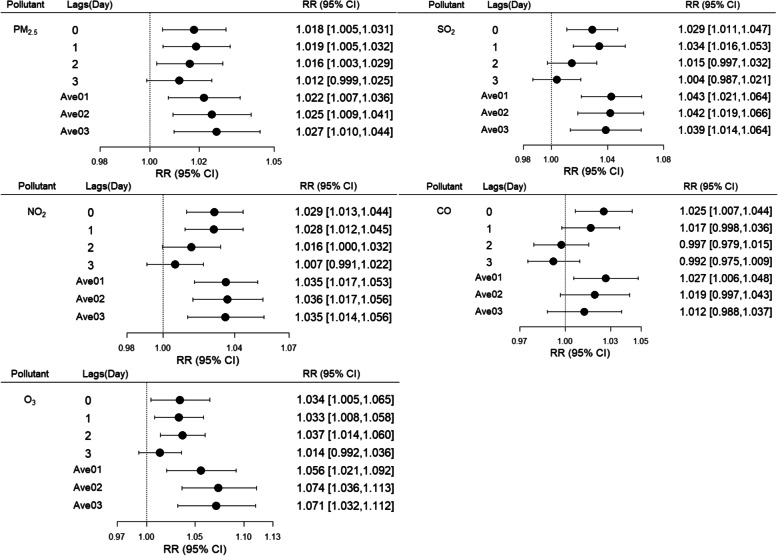
Associations between IQR increases in PM_2.5_, SO_2_, NO_2_, CO, and O_3_ and COPD-related mortality among elderly people at lag0, lag1, lag2, lag3, lag01, lag02, and lag03 days, respectively. All models were adjusted for daily temperature and relative humidity

**Fig. 3:**
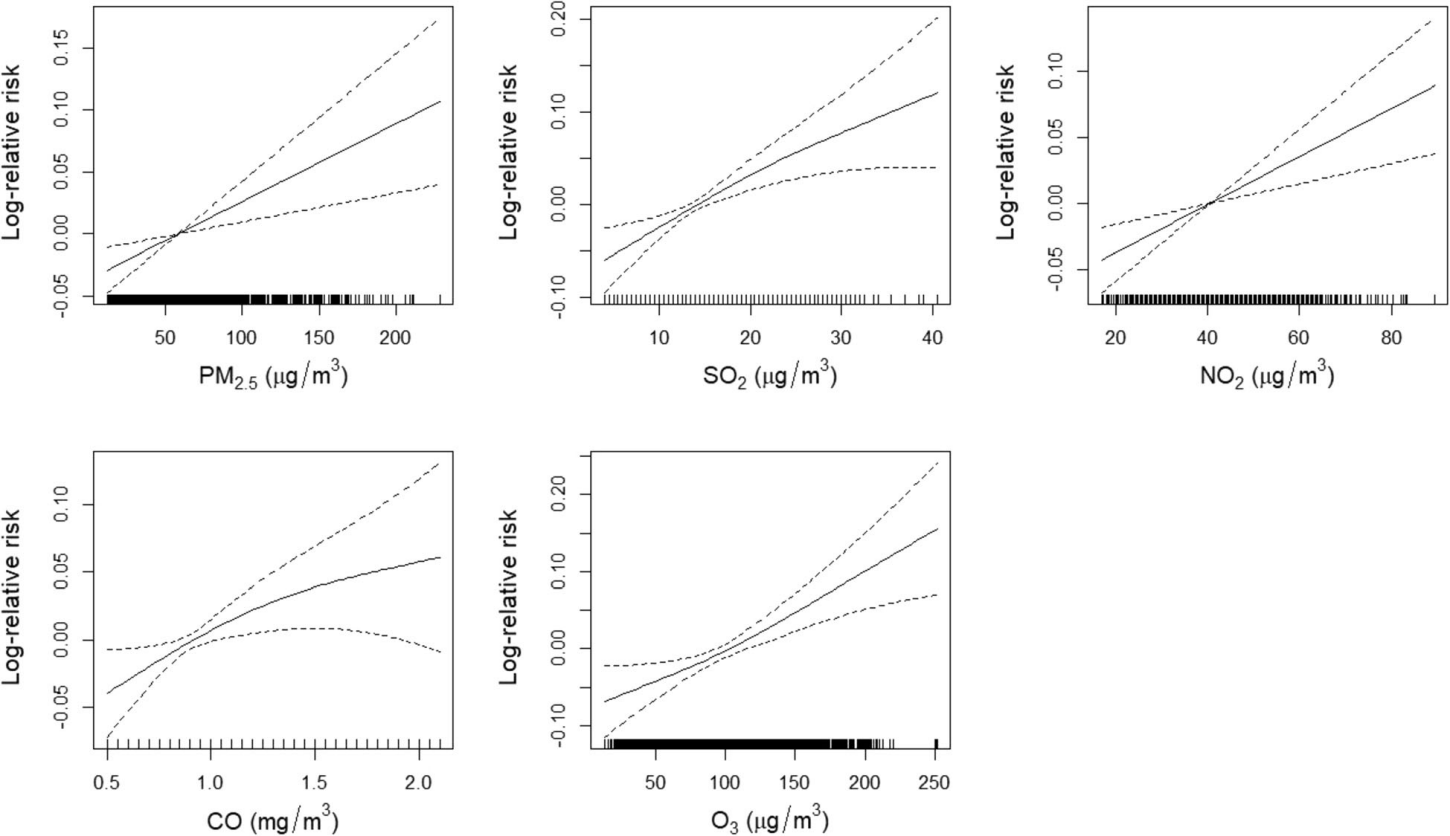
Exposure-response curves for associations between concentration increases in each ambient airborne pollutant and COPD-related mortality. Curves for PM_2.5_, SO_2_, NO_2_, CO, and O_3_ correspond to lag03, lag01, lag02, lag01, and lag02, respectively

Associations were apparent between COPD-related mortality and IQR increases in the concentrations of PM_2.5_ (43 μg/m^3^), SO_2_ (8 μg/m^3^), NO_2_ (18 μg/m^3^), CO (0.4 mg/m^3^), and O_3_ (78 μg/m^3^) after the influences of weather conditions (i.e., daily mean temperature and relative humidity) were controlled. The days corresponding to the greatest estimate results for PM_2.5_ (RR = 1.027, 95% CI 1.010–1.044), SO_2_ (RR = 1.043, 95% CI 1.021–1.064), NO_2_ (RR = 1.036, 95% CI 1.017–1.056), CO (RR = 1.027, 95% CI 1.006–1.048), and O_3_ (RR = 1.074, 95% CI 1.036–1.113) were lag03, lag01, lag02, lag01, and lag02 in the multi-day lag models, respectively. The estimate results calculated from the single-day lag models were similar, although a small amount lower than the results from the multi-day lag models for each pollutant (Fig. 2). The exposure-response curves exhibited linear relationships between each pollutant and the log-relative risk of COPD-related mortality (Fig. 3).

**Fig. 4:**
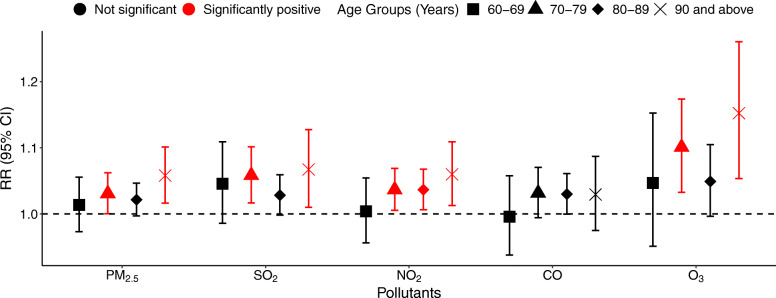
Associations between IQR increases in PM_2.5_, SO_2_, NO_2_, CO, and O_3_ and COPD-related mortality among different age groups

Statistically significant associations between COPD-related mortality and pollutants in the 60–69 age group were not observed, but were observed in the 70–79 and ≥ 90 age groups, with the exception of CO. The greatest effects of PM_2.5_, SO_2_, NO_2_, and O_3_ in the 70–79 age group were at lag03 (RR = 1.031, 95% CI 1.000–1.062), lag02 (RR = 1.058, 95% CI 1.017–1.102), lag01 (RR = 1.037, 95% CI 1.006–1.069), and lag02 (RR = 1.101, 95% CI 1.033–1.174), respectively, while in the ≥ 90 age group the corresponding effects were at lag03 (RR = 1.058, 95% CI 1.016–1.101), lag02 (RR = 1.067, 95% CI 1.010–1.128), lag02 (RR = 1.060, 95% CI 1.013–1.109), and lag03 (RR = 1.152, 95% CI 1.054–1.260), and the greatest effect of NO_2_ in the 80–89 age group was at lag03 (RR = 1.037, 95% CI 1.006–1.068). Statistically significant differences for the effects of airborne pollutants on COPD-related mortality were not observed between different age groups (Fig. 4).

The RRs for PM_2.5_, SO_2_, NO_2_, and O_3_ in males were 1.024 (95% CI 1.002–1.047), 1.048 (95% CI 1.017–1.079), 1.035 (95% CI 1.010–1.061), and 1.064 (95% CI 1.016–1.115), respectively, corresponding to lag03, lag02, lag02, and lag02. The RRs for PM_2.5_, SO_2_, NO_2_, and O_3_ in females were 1.030 (95% CI 1.005–1.056), 1.040 (95% CI 1.009–1.071), 1.039 (95% CI 1.013–1.065), and 1.087 (95% CI 1.029–1.149), respectively, corresponding to lag03, lag01, lag01, and lag03. The RRs for CO were not observed in either males or females. The RRs for PM_2.5_, SO_2_, NO_2_, CO, and O_3_ in married individuals were 1.033 (95% CI 1.010–1.057), 1.051 (95% CI 1.020–1.084), 1.048 (95% CI 1.019–1.078), 1.037 (95% CI 1.008–1.066), and 1.065 (95% CI 1.015–1.118), respectively, corresponding to lag03, lag02, lag03, lag01, and lag02. The RRs for PM_2.5_, SO_2_, and O_3_ in alternative marital status individuals (including divorced, widowed, and never married) were 1.020 (95% CI 1.000–1.041), 1.034 (95% CI 1.003–1.065), and 1.087 (95% CI 1.030–1.146), respectively, corresponding to lag01, lag01, and lag03. Statistically significant differences for the effects of airborne pollutants on COPD-related mortality were not observed between males and females, nor were they found between married individuals and alternative marital status individuals (Figure S2).

By adding the remaining pollutants each time as covariates into the models, two-pollutant model simulations were conducted for each pollutant corresponding to their greatest cumulative effect days. The effects of O_3_ remained steady after adjusting for PM_2.5_, SO_2_, NO_2_, and CO each time in the models. The effects of SO_2_ and NO_2_ attenuated after adjusting for each other. The effect of PM_2.5_ attenuated after adjusting for SO_2_ and NO_2_, respectively. The effects of CO attenuated after adjusting for PM_2.5_, SO_2_, and NO_2_, and O_3_ (Table 3).

**Table 3:** RRs for two-pollutant models including PM_2.5_, SO_2_, NO_2_, CO, and O_3_^b^

Airborne pollutant		RR	RR 95% CI	
			Lower	Upper
PM_2.5_		1.027 ^a^	1.010	1.044
	+SO_2_	1.017	0.995	1.039
	+NO_2_	1.013	0.986	1.039
	+CO	1.050 ^a^	1.023	1.078
	+O_3_	1.019 ^a^	1.001	1.037
SO_2_		1.043 ^a^	1.021	1.064
	+PM_2.5_	1.038 ^a^	1.010	1.067
	+NO_2_	1.024	0.993	1.057
	+CO	1.041 ^a^	1.015	1.068
	+O_3_	1.036 ^a^	1.014	1.058
NO_2_		1.036 ^a^	1.017	1.056
	+PM_2.5_	1.030 ^a^	1.000	1.061
	+SO_2_	1.022	0.993	1.052
	+CO	1.047 ^a^	1.020	1.074
	+O_3_	1.025 ^a^	1.005	1.046
CO		1.027 ^a^	1.006	1.048
	+PM_2.5_	1.004	0.972	1.038
	+SO_2_	1.002	0.976	1.028
	+NO_2_	0.993	0.964	1.023
	+O_3_	1.019	0.998	1.041
O_3_		1.074 ^a^	1.036	1.113
	+PM_2.5_	1.060 ^a^	1.021	1.101
	+SO_2_	1.057 ^a^	1.018	1.098
	+NO_2_	1.055 ^a^	1.016	1.097
	+CO	1.070 ^a^	1.032	1.110

Abbreviations: *PM*_*2.5*_ particulate matter < 2.5 μm in aerodynamic diameter, *SO*_*2*_ sulfur dioxide, *NO*_*2*_ nitrogen dioxide, *CO* carbon monoxide, *O*_*3*_ ozone, *RR* relative risk, *CI* confidence interval

^a^*P* < 0.05

^b^RRs for PM_2.5_, SO_2_, NO_2_, CO, and O_3_ were from lag03, lag01, lag02, lag01, and lag02, respectively

## Discussion

In this investigation, we employed a time-series study design to determine the associations between airborne pollutants and COPD-related mortality among elderly individuals aged 60 and above. After confounders consisting of daily mean temperature and relative humidity were controlled, IQR increases in the concentrations of PM_2.5_ (43 μg/m^3^), SO_2_ (8 μg/m^3^), NO_2_ (18 μg/m^3^), CO (0.4 mg/m^3^), and O_3_ (78 μg/m^3^) were associated with 2.7% (95% CI 1.0–4.4%), 4.3% (95% CI 2.1–6.4%), 3.6% (95% CI 1.7–5.6%), 2.7% (95% CI 0.6–4.8%), and 7.4% (95% CI 3.6–11.3%) increases in COPD-related mortality among elderly individuals aged 60 and above, respectively. The exposure-response curves for the aforementioned pollutants and COPD-related mortality were nearly linear, which is consistent with previous studies [20]. Some prior research reported adverse effects from gaseous pollutants composed of SO_2_, NO_2_, CO, and O_3_ on COPD-related mortality that were greater than that from fine particulates [21], while some reported the opposite [4, 22]. In our study, COPD-related mortality exhibited stronger associations with gaseous pollutants than with fine particulates.

The adverse effects of PM_2.5_, SO_2_, NO_2_, CO, and O_3_ on COPD-related mortality in our study were significantly greater than those in previous studies. A nationwide study including 272 cities in China for the period 2013–2015 assessed associations between ambient airborne pollutants comprised of PM_2.5_, SO_2_, NO_2_, and O_3_ and daily COPD mortality [23–26], in which 10 μg/m^3^ increases in PM_2.5,_ SO_2_, and NO_2_ concentrations were associated with 0.38%, 0.69%, and 1.6% increases in COPD mortality, respectively, while the effect of O_3_ was not significant. A study of four Chinese cities revealed that 10 μg/m^3^ increases in PM_10_, SO_2_, and NO_2_ concentrations were related to 0.78, 1.38, and 1.85% increases in COPD mortality, respectively [20]. A case-crossover study in Shanghai indicated that 10 μg/m^3^ increases in PM_10_, SO_2_, and NO_2_ concentrations could increase COPD mortality risks by 0.6, 3.3, and 4.2%, respectively [27]. In a time series study in the Netherlands from 1992–2006, the excess risks of COPD mortality due to the effects of 10 μg/m^3^ increases in the concentrations of PM_2.5_, NO_2_, and O_3_ were 0.9, 0.7, and 0.5%, respectively (16). An extended follow-up of the Harvard Six Cities Study found non-statistically significant associations between PM_2.5_ and COPD mortality [28]. The estimate results of our study were substantially higher than the results from the aforementioned research, despite the fact that the individuals in our study were aged 60 and older, while the results in previous studies were usually based on the entire population.

Although no statistically significant differences were observed among different age groups, the very elderly individuals might be more susceptible to COPD-related mortality due to air pollution than the younger elderly in our study. Some previous investigations consistently reported negative results for the influence of sociodemographic factors, including age and gender [23, 25, 26, 29]. At the same time, some studies reported that airborne pollutants exerted larger impacts on COPD in males than in females [9, 22, 30], raising the suspicion that smoking plays a synergistic role that enhances the effects of airborne pollutants. Therefore, future research is still needed on the modified effects of sociodemographic factors, including age and gender, with regard to air pollution and COPD-related mortality.

The associations of SO_2_ and NO_2_ with COPD-related mortality became non-statistically significant after adjusting for each other in our study, with some previous research reporting similar results [31, 32]. There were also probable interactions between SO_2_ and NO_2_. Conversely, O_3_ exerted very stable influences on COPD-related mortality in our study, which was in accordance with previous research [33, 34]. Adjusting for the remaining airborne pollutants did not alter the values of the effect estimates for O_3_, suggesting that O_3_ might be a more important exposure indicator than other ambient airborne pollutants when it comes to ambient air pollution and COPD-related mortality. The effects of PM_2.5_ were attenuated after adjusting for NO_2_, but were intensified after adjusting for CO; the effects of NO_2_ were intensified after adjusting for either PM_2.5_ or CO; and the effects of CO were attenuated after adjusted for either PM_2.5_ or NO_2_. Considering the high correlations among PM_2.5_, NO_2_, and CO, there might be potential collinearity among them. Moreover, CO exhibited unstable adverse effects in our study. After adjusting for the remaining pollutants each time, the effects of CO were attenuated. Previous studies also reported unstable associations between ambient CO and COPD occurrences, hospitalizations, and emergency visits [35–37]. The effects of ambient CO on COPD-related mortality require further confirmation in future research.

Ambient airborne pollutants mostly originate from industrial emissions, traffic emissions, and the combustion of fuel [38–40]. The concentrations of airborne pollutants exhibited typical seasonal tendencies in our study. The concentrations of PM_2.5_, SO_2_, NO_2_, and CO were relatively high during the winter and lower during the summer. This was probably due to the reduction of airflow in winter and increased fuel consumption for heat. The concentration of O_3_ was significantly higher in summer, which may be due to the increased sunlight. The influences of the seasonal tendencies were controlled using a natural cubic spline function in the models.

Airborne pollutants, including fine particulate matter and gaseous pollutants, could induce airway cellular injuries and cause airway epithelial cell apoptosis, which stimulates airway inflammation [41–43]. Progressive inflammatory disease in airways, alveoli, and microvasculature could induce COPD [44]. This represents a probable mechanism for airborne pollutants exacerbating COPD-related mortality. Another potential explanation is that individuals with COPD may be more susceptible to the acute cardiopulmonary effects of airborne pollutant exposure, thereby resulting in the higher risk of death [45].

The robustness of the models was tested using sensitivity analyses. The RRs calculated via different degrees of freedom for time trends ranging from 5 to 9 in the models were similar (Table S1). Hence, the results calculated from the models were reliable.

This study has three main strengths. First, it is the first to utilize a time-series study to examine the associations between ambient airborne pollutants and COPD-related mortality in the Sichuan Basin, located in the hinterland of southwestern China. Second, the mortality data were derived from the PDIRMS and were thus authentic and reliable. Third, the large and dense population and comparatively poor air conditions of Chengdu city made the area advantageous for conducting such an environmental study of the adverse effects of airborne pollutants on health. There are also some limitations, however. The exposure data were derived from 23 fixed monitoring sites, while personal exposure data were not obtained.

## Conclusions

This study found that the ambient airborne pollutants PM_2.5_, SO_2_, NO_2_, O_3_, and CO were associated with COPD-related mortality in the central Sichuan Basin, located in the hinterland of southwestern China. The adverse effects of O_3_ were stable. This study adds limited evidence to air pollution and health outcomes. Further research on personal exposure and the components of the particulate matter is needed to more accurately analyze air pollution and COPD-related mortality.

## Supplementary Information

**Additional file 1: Figure S1.** Seasonal trends of daily mean concentrations of PM_2.5_, SO_2_, NO_2_, CO, and daily 8-hour mean concentrations of O_3_. **Figure S2.** Associations between IQR increases in PM_2.5_, SO_2_, NO_2_, CO, and O_3_ and COPD-related mortality between different genders, and between different marital statuses.

**Additional file 2. Table S1.** RRs via alternative degrees of freedom (DFs) for time trends ranging from 5 to 9 in the models

## Data Availability

The datasets used in this study are available from the corresponding author upon reasonable request.

## Abbreviations

PM_2.5_: Particulate matter < 2.5 μm in aerodynamic diameter
SO_2_: Sulfur dioxide
NO_2_: Nitrogen dioxide
CO: Carbon monoxide
O_3_-8h: Daily 8-h mean concentration of ozone
RH: Relative humidity
COPD: Chronic obstructive pulmonary disease
SD: Standard deviation
IQR: Interquartile range
RR: Relative risk
CI: Confidence interval
