# Correction to: Effects of short-term exposure to ambient airborne pollutants on COPD-related mortality among the elderly residents of Chengdu city in Southwest China

**DOI:** 10.1186/s12199-021-00947-z

**Published:** 2021-02-16

**Authors:** Jianyu Chen, Chunli Shi, Yang Li, Hongzhen Ni, Jie Zeng, Rong Lu, Li Zhang

**Affiliations:** Sichuan Provincial Center for Disease Control and Prevention, No. 6, Zhongxue Road, Wuhou District, Chengdu, 610041 People’s Republic of China

**Correction to: Environ Health Prev Med (2021) 26:7**

**https://doi.org/10.1186/s12199-020-00925-x**

Following publication of the original article [1], the authors noticed that only one corresponding author was published online. In the submitted manuscript, there are two corresponding authors, Jianyu Chen and Li Zhang, but only Jianyu Chen was reserved in the published version.

The original article [1] has been updated.
