# Supplementary figures and images for: Effects of short-term exposure to ambient airborne pollutants on COPD-related mortality among the elderly residents of Chengdu city in Southwest China
Source: Environ Health Prev Med. 2021 Jan 12;26:7. doi: 10.1186/s12199-020-00925-x (PMC7805042; doi:10.1186/s12199-020-00925-x)

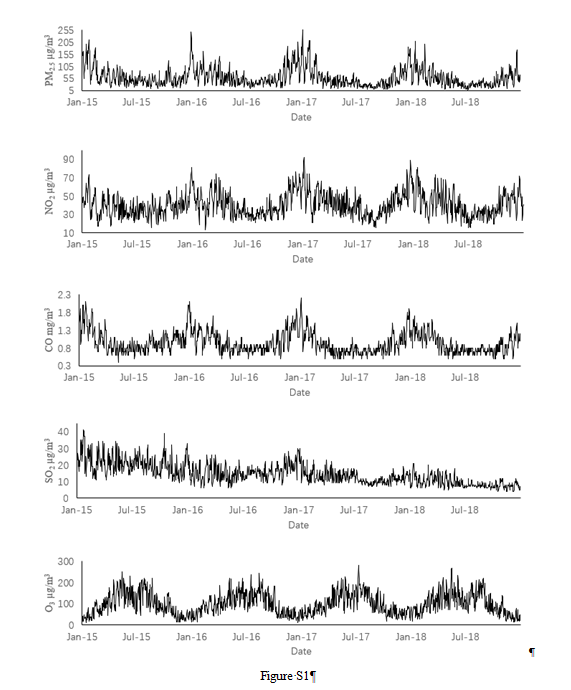


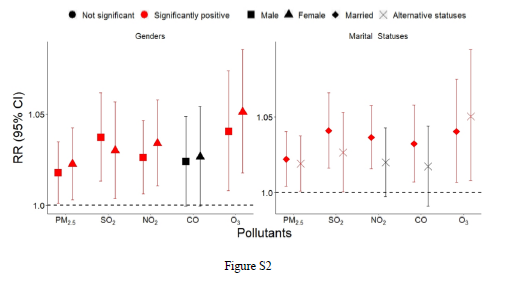

Supplement: Supplementary file 1 — Additional file 1: Figure S1. Seasonal trends of daily mean concentrations of PM2.5, SO2, NO2, CO, and daily 8-hour mean concentrations of O3. Figure S2. Associations between IQR increases in PM2.5, SO2, NO2, CO, and O3 and COPD-related mortality between different genders, and between different marital statuses. [file 12199_2020_925_MOESM1_ESM.docx]
