# Supplementary material for: Effects of short-term exposure to ambient airborne pollutants on COPD-related mortality among the elderly residents of Chengdu city in Southwest China
Source: Environ Health Prev Med. 2021 Jan 12;26:7. doi: 10.1186/s12199-020-00925-x (PMC7805042; doi:10.1186/s12199-020-00925-x)
Supplement: Supplementary file 2 — Additional file 2. Table S1. RRs via alternative degrees of freedom (DFs) for time trends ranging from 5 to 9 in the models [file 12199_2020_925_MOESM2_ESM.docx]

Table S1. RRs via alternative degrees of freedom (DFs) for time trends ranging from 5 to 9 in the models

| Airborne pollutant | RR | RR 95% CI | | | Lags (day) | DFs |
| --- | --- | --- | --- | --- | --- | --- |
|  |  |  | Lower | Upper |  |  |
| PM_2.5_ | 1.029 ^a^ |  | 1.015 | 1.044 | 01 | 5 |
|  | 1.034 ^a^ |  | 1.018 | 1.050 | 02 | 5 |
|  | 1.038 ^a^ |  | 1.021 | 1.055 | 03 | 5 |
|  | 1.025 ^a^ |  | 1.011 | 1.040 | 01 | 6 |
|  | 1.029 ^a^ |  | 1.014 | 1.045 | 02 | 6 |
|  | 1.032 ^a^ |  | 1.015 | 1.049 | 03 | 6 |
|  | 1.022 ^a^ |  | 1.007 | 1.036 | 01 | 7 |
|  | 1.025 ^a^ |  | 1.009 | 1.041 | 02 | 7 |
|  | 1.026 ^a^ |  | 1.010 | 1.044 | 03 | 7 |
|  | 1.020 ^a^ |  | 1.005 | 1.035 | 01 | 8 |
|  | 1.023 ^a^ |  | 1.007 | 1.039 | 02 | 8 |
|  | 1.024 ^a^ |  | 1.007 | 1.042 | 03 | 8 |
|  | 1.020 ^a^ |  | 1.005 | 1.035 | 01 | 9 |
|  | 1.023 ^a^ |  | 1.007 | 1.039 | 02 | 9 |
|  | 1.024 ^a^ |  | 1.007 | 1.042 | 03 | 9 |
| SO_2_ | 1.042 ^a^ |  | 1.021 | 1.064 | 01 | 5 |
|  | 1.041 ^a^ |  | 1.018 | 1.065 | 02 | 5 |
|  | 1.037 ^a^ |  | 1.012 | 1.063 | 03 | 5 |
|  | 1.040 ^a^ |  | 1.019 | 1.062 | 01 | 6 |
|  | 1.039 ^a^ |  | 1.016 | 1.063 | 02 | 6 |
|  | 1.036 ^a^ |  | 1.011 | 1.061 | 03 | 6 |
|  | 1.043 ^a^ |  | 1.021 | 1.064 | 01 | 7 |
|  | 1.042 ^a^ |  | 1.019 | 1.066 | 02 | 7 |
|  | 1.039 ^a^ |  | 1.014 | 1.064 | 03 | 7 |
|  | 1.042 ^a^ |  | 1.020 | 1.064 | 01 | 8 |
|  | 1.041 ^a^ |  | 1.017 | 1.065 | 02 | 8 |
|  | 1.037 ^a^ |  | 1.011 | 1.063 | 03 | 8 |
|  | 1.042 ^a^ |  | 1.020 | 1.064 | 01 | 9 |
|  | 1.041 ^a^ |  | 1.017 | 1.065 | 02 | 9 |
|  | 1.037 ^a^ |  | 1.011 | 1.063 | 03 | 9 |
| NO_2_ | 1.032 ^a^ |  | 1.015 | 1.050 | 01 | 5 |
|  | 1.033 ^a^ |  | 1.014 | 1.053 | 02 | 5 |
|  | 1.032 ^a^ |  | 1.011 | 1.053 | 03 | 5 |
|  | 1.034 ^a^ |  | 1.017 | 1.052 | 01 | 6 |
|  | 1.035 ^a^ |  | 1.016 | 1.053 | 02 | 6 |
|  | 1.035 ^a^ |  | 1.014 | 1.056 | 03 | 6 |
|  | 1.035 ^a^ |  | 1.017 | 1.053 | 01 | 7 |
|  | 1.036 ^a^ |  | 1.017 | 1.056 | 02 | 7 |
|  | 1.035 ^a^ |  | 1.014 | 1.056 | 03 | 7 |
|  | 1.035 ^a^ |  | 1.017 | 1.053 | 01 | 8 |
|  | 1.036 ^a^ |  | 1.016 | 1.056 | 02 | 8 |
|  | 1.034 ^a^ |  | 1.012 | 1.056 | 03 | 8 |
|  | 1.035 ^a^ |  | 1.017 | 1.053 | 01 | 9 |
|  | 1.036 ^a^ |  | 1.016 | 1.056 | 02 | 9 |
|  | 1.034 ^a^ |  | 1.012 | 1.056 | 03 | 9 |
| CO | 1.039 ^a^ |  | 1.018 | 1.060 | 01 | 5 |
|  | 1.034 ^a^ |  | 1.011 | 1.057 | 02 | 5 |
|  | 1.029 ^a^ |  | 1.005 | 1.054 | 03 | 5 |
|  | 1.031 ^a^ |  | 1.010 | 1.053 | 01 | 6 |
|  | 1.025 ^a^ |  | 1.002 | 1.048 | 02 | 6 |
|  | 1.019 |  | 0.995 | 1.043 | 03 | 6 |
|  | 1.027 ^a^ |  | 1.006 | 1.048 | 01 | 7 |
|  | 1.019 |  | 0.997 | 1.043 | 02 | 7 |
|  | 1.012 |  | 0.988 | 1.037 | 03 | 7 |
|  | 1.024 ^a^ |  | 1.003 | 1.046 | 01 | 8 |
|  | 1.016 |  | 0.993 | 1.040 | 02 | 8 |
|  | 1.009 |  | 0.984 | 1.034 | 03 | 8 |
|  | 1.024 ^a^ |  | 1.003 | 1.046 | 01 | 9 |
|  | 1.016 |  | 0.993 | 1.040 | 02 | 9 |
|  | 1.009 |  | 0.984 | 1.035 | 03 | 9 |
| O_3_ | 1.045 ^a^ |  | 1.010 | 1.081 | 01 | 5 |
|  | 1.060 ^a^ |  | 1.022 | 1.098 | 02 | 5 |
|  | 1.055 ^a^ |  | 1.017 | 1.095 | 03 | 5 |
|  | 1.051 ^a^ |  | 1.016 | 1.088 | 01 | 6 |
|  | 1.069 ^a^ |  | 1.032 | 1.108 | 02 | 6 |
|  | 1.068 ^a^ |  | 1.029 | 1.109 | 03 | 6 |
|  | 1.056 ^a^ |  | 1.021 | 1.092 | 01 | 7 |
|  | 1.074 ^a^ |  | 1.036 | 1.113 | 02 | 7 |
|  | 1.071 ^a^ |  | 1.032 | 1.112 | 03 | 7 |
|  | 1.053 ^a^ |  | 1.018 | 1.089 | 01 | 8 |
|  | 1.071 ^a^ |  | 1.033 | 1.110 | 02 | 8 |
|  | 1.068 ^a^ |  | 1.028 | 1.109 | 03 | 8 |
|  | 1.053 ^a^ |  | 1.018 | 1.089 | 01 | 9 |
|  | 1.071 ^a^ |  | 1.033 | 1.110 | 02 | 9 |
|  | 1.068 ^a^ |  | 1.028 | 1.109 | 03 | 9 |

Abbreviations: PM_2.5_, particulate matter < 2.5 μm in aerodynamic diameter; SO_2_, sulfur dioxide; NO_2_, nitrogen dioxide; CO, carbon monoxide; O_3_, ozone; RR, relative risk; CI, confidence interval.

^a^ *P* < 0.05.
